# Probing the orientation of electrostatically immobilized cytochrome C by time of flight secondary ion mass spectrometry and sum frequency generation spectroscopy

**DOI:** 10.1186/1559-4106-8-18

**Published:** 2013-07-26

**Authors:** Joe E Baio, Tobias Weidner, Dennis Ramey, Leah Pruzinsky, David G Castner

**Affiliations:** 1 National ESCA and Surface Analysis Center for Biomedical Problems, Department of Chemical Engineering, University of Washington, Seattle, USA; 2 National ESCA and Surface Analysis Center for Biomedical Problems, Department of Bioengineering, University of Washington, Seattle, USA; 3 Department of Biomedical Engineering, University of Connecticut, Storrs, CT, USA; 4 School of Chemical, Biological and Environmental Engineering, Oregon State University, Corvallis, OR, USA; 5 Max Planck Institute for Polymer Research, Mainz, Germany

## Abstract

By taking advantage of the electron pathway through the heme group in cytochrome c (CytoC) electrochemists have built sensors based upon CytoC immobilized onto metal electrodes. Previous studies have shown that the electron transfer rate through the protein is a function of the position of this heme group with respect to the electrode surface. In this study a detailed examination of CytoC orientation when electrostatically immobilized onto both amine (NH_3_^+^) and carboxyl (COO^-^) functionalized gold is presented. Protein coverage, on both surfaces, was monitored by the change in the atomic % N, as determined by x-ray photoelectron spectroscopy. Spectral features within the *in situ* sum frequency generation vibrational spectra, acquired for the protein interacting with positively and negatively charged surfaces, indicates that these electrostatic interactions do induce the protein into a well ordered film. Time of flight secondary ion mass spectrometry data demonstrated a clear separation between the two samples based on the intensity differences of secondary ions stemming from amino acids located asymmetrically within CytoC (cysteine: C_2_H_6_NS^+^; glutamic acid: C_4_H_6_NO^+^ and C_4_H_8_NO_2_^+^; leucine: C_5_H_12_N^+^). For a more quantitative examination of orientation, we developed a ratio comparing the sum of the intensities of secondary-ions stemming from the amino acid residues at either end of the protein. The 50 % increase in this ratio, observed between the protein covered NH_3_^+^ and COO^-^ substrates, indicates opposite orientations of the CytoC on the two different surfaces.

## Background

The heme group within cytochrome C (CytoC) provides an essential pathway for electrons traveling across a cell membrane [1]. Taking advantage of this electron transfer pathway electrochemists have developed a series of devices based on CytoC immobilized onto both modified metal oxides [2–4] and alkanethiol SAMs [5–7]. Combined this work has demonstrated that the electron transfer rate through the protein to the electrode is directly related to the distance between the heme group and the substrate [8]. Therefore, the distance between the heme group and electron transfer pathway can be tuned by altering the protein’s orientation.

Some of the most detailed characterizations of surface immobilized CytoC have been published by the Saavedra group. In a series of papers Saavedra and coworkers tracked the orientation of the heme group, within the protein, with linear dichroism in combination with emission anisotropy measured in total internal reflection fluorescence (TIRF) [9]. This TIRF work has led to a detailed library of mean heme tilt angles for CytoC immobilized onto SH-, OH-, CH_3_-, and SO_3_-SAMs [10–14]. However, this TIRF work only provides details about the tilt angle of the heme group within the protein and not any information about the overall oreintation of the protein. As multiple CytoC orientations could produce similar heme tilt angles, focusing only on the heme tilt angle may not provide the distance between the heme group and the electrode.

**Figure 1:**
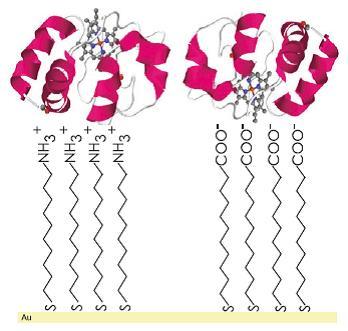
**Schematic diagram of CytoC immobilized via electrostatic interactions onto positively (NH**_**2**_**-SAMs) and negatively (COOH-SAMs) charged substrates** [17]**.**

Electrochemical measurements of CytoC immobilized onto Au electrodes modified by COOH- and NH_2_-SAMs reveal a large shift in the electron transfer rate between the heme and electrode as you switch the immobilization scheme [7]. When immobilized onto a charged surface, CytoC, with its distribution of lysine and glutamate residues around its surface, should orient and form a well-ordered protein film [15–17]. To examine how this dipole like distribution of charge influences the orientation of the whole protein, Xu *et al.* tracked the reactivity of lysines around the heme domain of CytoC adsorbed onto COOH-SAMs [6]. This technique assumes that in a well-ordered protein film the reactivity of the lysine groups will be a function of their accessibility. Xu *et al.* reported that the least reactive lysine residues were located around the heme interface [6]. A result that implies that on a negatively charged surface the protein is oriented with the heme group pointed down towards the electrode. Conversely, when CytoC interacts with a positively charged surface (*e.g*. NH_2_-SAMs), this same dipole like distribution of charge should induce the exact opposite orientation (Figure 1), resulting in a change of the distance between the heme and the electrode.

To test this hypothesis while also connecting previous electrochemical observations to CytoC’s structure, ordering and orientation, we electrostatically immobilized CytoC onto positively and negatively charged substrates then we characterized differences in protein orientation by time of flight secondary ion mass spectrometry (ToF-SIMS), as well as probed protein ordering by sum frequency generation vibrational spectroscopy (SFG). Defining the ordering and orientation of the protein at the electrode surface enabled us to characterize any change in distance between the heme group and the electrode.

ToF-SIMS and SFG are two surface analysis techniques that provide complementary information about surface bound proteins. Taking advantage of ToF-SIMS’ high mass resolution (>4000 m/Δm) and excellent surface sensitivity (~2 nm sampling depth) changes in conformation and orientation of relatively thick protein films (>10 nm) can be directly related to changes in the intensity of specific secondary ions [18–28]. Recently two model systems built upon a small covalently and electrostatically immobilized protein (B1 domain of Protein G, 6 kDa), illustrated that differences in orientation can be discerned through the comparison of ratios of intensities of secondary ions originating from amino acid residues at opposite ends of the protein [29, 30]. These model systems also proved that ToF-SIMS can determine protein orientation even when the thickness of the protein is similar to the SIMS sampling depth.

To complement the protein orientation information provided by ToF-SIMS, conformational changes and the ordering of specific secondary structures (α-helices, β-sheets and β-turns) can be assessed by probing amide, N-H, C-H, and O-H vibrational modes with SFG [31–38] Additionally, tilt angles of helicies within the protein can be calculated from the polarization dependence of the SFG amide peaks [39, 40].

## Methods

### Functionalized substrates

1×1 cm^2^ silicon substrates (Microelectronics Inc., San Jose, CA) were cleaned by sequential sonication in deionized water, dichloromethylene, acetone, and methanol. In a high vacuum electron beam evaporator (pressure < 1×10^-6^ Torr), substrates were then coated with a thin layer of titanium (10 nm) followed by high purity gold (99.99%, 80 nm). Following established protocols [41], NH_2_-SAMs were assembled by submerging the Au coated substrates into a 0.5 mM 11-amino-1-undecanthiol, hydrochloride (Asemblon, Redmond, WA) ethanol solution. The substrates were allowed to soak in this ethanol–thiol solution for at least 16 h. Following assembly, the substrates were removed from solution, rinsed with pure ethanol, and then vortexed within a 10% v/v NH_4_OH ethanol solution. COOH-SAMs were assembled by soaking Au substrates in a 1-mM 11-mercaptoundecanoic acid (Aldrich, St. Louis, MO) ethanol solution for at least 16 h and then sonicated within pure ethanol. Following the rinse step, all substrates were dried and stored under nitrogen until analysis or protein adsorption.

### Protein immobilization

Horse heart cytochrome c (12 kDa) was purchased commercially (Sigma, St. Louis, Missouri). For protein immobilization, the NH_2_- and COOH substrates were submerged in an ultra low salt phosphate buffered saline protein solution (1.37 mM NaCl, 2.7 mM KCl, 10 mM sodium phophate, and 2 mM potassium phosphate pH 6 and 8, respectively) at CytoC concentrations of 5, 25, 100, and 200 μM to produce surfaces with varying degrees of protein coverage. The two different pH’s were chosen to maximize the charge on the substrate [42] while maintaining both positive and negative charges within the protein (CytoC’s pI = 10) [43]. Binding proceeded for 1 h at room temperature. Following protein immobilization, samples were washed by serial dilution in buffer, followed by submerging samples in a series of stirred water solutions. Samples were then air-dried and stored under nitrogen.

### X-ray photoelectron spectroscopy (XPS)

XPS compositions were determined from an average of three spots on two replicate samples. The data were acquired on a Kratos AXIS Ultra DLD instrument (Kratos, Manchester, England) in the hybrid mode using a 0° take-off angle (angle between the surface normal and the axis of the analyzer lens) and a monochromatic Al Kα_1,2_ x-ray source (*hv* = 1486.6 eV). Atomic compositions were calculated from C 1 s and Au 4f peak areas obtained from survey scans (0–1100 eV) plus O 1 s (524–544 eV), N 1 s (390–410 eV), and S 2p (155–173 eV) peak areas from narrow scans (analyzer pass energy = 80 eV for both survey and narrow scan spectra). Energy scales were calibrated by setting the large CH_*x*_ peak in the C 1 s region to 285.0 eV and a linear background was subtracted for all peak area quantifications. The peak areas were normalized by the manufacturer supplied sensitivity factors and surface concentrations were calculated using CASA XPS (Casa Software Ltd).

The amount of protein on the surface was tracked by the nitrogen atomic percent (At %) determined from the N 1 s signal. The NH_2_-SAM substrates contain nitrogen, therefore, by examining the attenuation of the Au 4f signal from the Au substrate before and after protein immobilization the nitrogen contribution from just the protein layer can be calculated by Equation 1 [25, 29].1
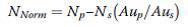


Where, N_s_ and Au_s_ are the measured N and Au At %, respectively, from the NH_2_ SAM covered Au substrate prior to protein immobilization; N_p_ and Au_p_ are the measured N and Au At %, respectively, from the NH_2_ SAM covered Au substrate after protein immobilization; and N_norm_ is the N At % corresponding to just the protein layer.

### Time-of-flight secondary ion mass spectrometry (ToF-SIMS)

Positive secondary ion spectra were acquired on a TOF.SIMS 5–100 instrument (ION-TOF, Munster, Germany) using a pulsed 25 keV Bi_3_^+^ primary ion beam under static conditions (primary ion dose < 10^12^ ions/cm^2^). Spectra were collected from three 100×100 μm^2^ regions per sample. Secondary ions were collected over a range of 0–800 m/z at a mass resolution (*m/Δm*) between 4000 and 8000. Spectra were mass calibrated using CH_2_^+^, C_2_H_2_^+^, C_3_H_5_^+^ and AuSCH_2_^+^ peaks. Mass calibration errors were typically below 20 ppm.

### Sum frequency generation spectroscopy (SFG)

SFG spectra were obtained by overlapping fixed visible (532 nm) and tunable IR (1000 – 4000 cm^-1^) pulses, in time and space, using an EKSPLA Nd:YAG laser operating at 50 Hz that also pumped an EKSPLA optical parametric generation/amplification and difference frequency unit based on barium borate and AgGaS_2_ crystals. The IR light had a bandwidth of 4 cm^-1^ and the visible light had a bandwidth of 1 cm^-1^. Both beams were focused to a diameter of ~1 mm at the sample surface at an energy of 160 μJ per pulse. All vibrational spectra were collected at 4 cm^-1^ increments and the generated SFG signal was first spectrally filtered and dispersed by a monochromator, then detected with a gated photomultiplier tube.

SFG spectra were collected at two different polarization combinations ssp (s-polarized SFG signal, s-polarized input visible, and p-polarized input IR beam) and ppp (p-polarized SFG signal, p-polarized input visible, and p-polarized input IR beam) through the backside of a CaF_2_ prism partially submerged into a protein buffer solution (protein concentration was 100 μM). A bare CaF_2_ prism was used as a model positively charged substrate while a negatively charged surface was created by oxygen plasma treating a CaF_2_ prism coated with polystyrene. The resulting signals were normalized by a reference signal generated in a ZnS crystal. The fitting routine used for SFG data analysis has been described previously [44].

## Results and discussion

In this study horse heart CytoC was immobilized onto positively and negatively charged substrates to induce opposite end-on orientations of CytoC, as done previously for Protein G B1 [29]. The two different CytoC orientations are expected because of the distribution of positively charged lysine residues distributed about the protein surface and the collection of negatively charged glutamic acids at one end of the protein [15–17].

**Figure 2:**
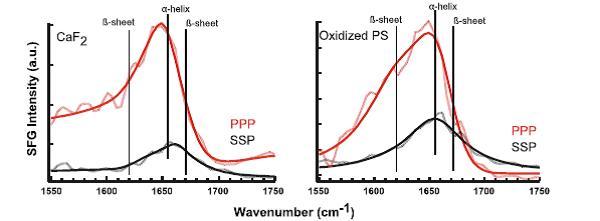
***In situ*SFG amide I spectra of CytoC interacting with a CaF**_**2**_**prism (left panel) and a CaF**_**2**_**prism coated with an oxygen plasma treated polystyrene (right panel).** Spectra were collected at two different polarization combinations ssp (black) and ppp (red). The amide peak near 1655 cm^-1^ is characteristic of ordered α-helices.

To examine the degree of ordering as this protein is immobilized onto a charged surface, we collected SFG data of CytoC interacting (protein solution concentration =100 μM), *in situ*, with both positively (CaF_2_ prism) and negatively (oxygen plasma treated polystyrene) charged surfaces with ssp and ppp polarization combinations. Comparing the intensities of spectral features across different polarization combinations (i.e. ppp versus ssp) provides a qualitative view of orientation. The SFG amide I spectra of the protein interacting with both surfaces are shown in Figure 2. Within this amide stretching region is a vibrational mode at 1655 cm^-1^ that is characteristic of ordered α-helices [36, 38, 40], and two peaks at 1620 and 1670 cm^-1^ related to ordered β-sheets [39]. CytoC contains several helical structures and the peak at 1655 cm^-1^ represents the average direction of the corona of helicies surrounding the heme group. Unlike other vibrational spectroscopies, the SFG selection rules dictate that these amide vibrational modes will only start to appear if secondary structures within the protein are ordered at the interface. For both substrates the intensity of this α-helical mode was significantly lower in the ssp polarization combination. This is indicative of a protein film that absorbs onto the surface with uniform orientations [22, 29, 30]. Ideally, this change in intensity can provide a quantitative picture (i.e. tilt-angles of protein secondary structure with respect to the substrate) of well-ordered protein films. Unfortunately, we were not able to calculate an average tilt angle for alpha helices using the method of Nguyen *et al.*, which suggests the helices in CytoC have a multi-modal distribution of orientations [40].

Overall, the SFG spectra provide a qualitative view of ordering and indicate that the electrostatic interactions between the protein and the substrates guide the adsorption of a well-ordered protein film. However, these results do not provide any detail about the structure and orientation of the protein. Electrostatically immobilizing CytoC onto positively (NH_2_-SAMs) and negatively charged (COOH-SAMs) substrates should induce the protein into opposite end-on orientations, thereby, changing the distance between the heme group and the substrate surface (Figure 1).

As in previous protein immobilization studies, differences in orientation were probed using ToF-SIMS by monitoring the intensity of specific secondary ions stemming from amino acid residues at either end of the protein [18, 22, 29, 30]. Here we apply this same strategy to probe any differences in CytoC orientation induced by the positively and negatively charged substrates (Figure 1).

**Figure 3:**
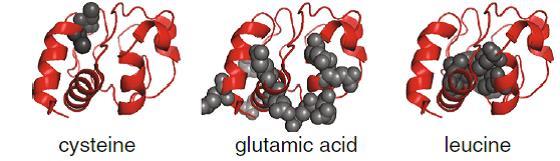
**Amino acids with asymmetric distributions used in the ToF-SIMS analysis are shown in grey** [17]**.**

Recent studies have shown that despite the complexity of ToF-SIMS data, the analysis can be reduced by just examining the ratio of the sum of intensities of secondary ions asymmetrically located in opposite ends of the protein. In the case of CytoC there is a cysteine rich region next to the heme group and a glutamic acid and leucine rich region at the opposite end of the protein (Figure 3). As a result of the shallow sampling depth of SIMS, the relative intensities of secondary-ions that originate from cysteine (C_2_H_6_NS^+^), glutamic acid (C_4_H_6_NO^+^, C_4_H_8_NO_2_^+^) and leucine (C_5_H_12_N^+^) (Table 1) should vary with protein orientation [45].

**Table 1:** Secondary ion fragments used in the ToF-SIMS analysis

Source	Formula	Mass
Cysteine	C_2_H_6_NS^+^	76.0351
Glutamic Acid	C_4_H_6_NO^+^	84.0526
	C_4_H_8_NO_2_^+^	102.0638
Leucine/Isoleucine	C_5_H_12_N^+^	86.1061

None of the four secondary ions listed above are present in spectra taken from the NH_2_- and COOH-SAMs prior to CytoC immobilization.

**Figure 4:**
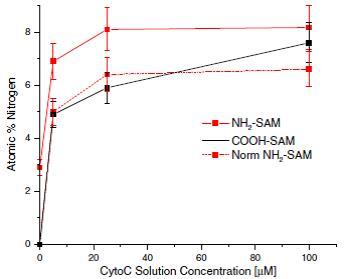
**Nitrogen concentrations determined by XPS analysis for CytoC immobilized onto NH**_**2**_**-SAMs (total nitrogen = solid red; normalized nitrogen = dashed red) or on COOH-SAMs (total nitrogen = solid black).** Data points represent average N concentration from six analysis spots on two different samples. Error bars represent standard deviations.

To minimize concentration based matrix and orientation effects it is best to compare secondary ion ratios between samples with similar protein coverages [46]. The protein surface concentration can be tracked by the XPS determined At % nitrogen. For the CytoC-NH_2_-SAM case, the nitrogen concentration at the surface increases with increasing solution concentration until the isotherm starts to plateau at 25 μM. At a CytoC solution concentration of 100 μM, both the normalized CytoC-NH_2_-SAM and the CytoC-COOH-SAM samples exhibit similar amounts of nitrogen, 6.6 ± 0.6 At % versus 7.6 ± 0.3 At % (Figure 4; Table 2) [22, 29]. Therefore, we only compared ToF-SIMS data taken from samples created at solution concentrations of 100 μM. This was also the same protein solution concentration used during the SFG experiments. Here we are assuming that adsorption is driven by electrostatic interactions – therefore, we do not expect the surface concentrations to be drastically different across techniques.

**Table 2:** **Summary of XPS determined elemental composition (atom %) for NH**_**2**_**-SAMs and COOH-SAMs before and after CytoC adsorption**

	At %					
Sample	Au 4f	C 1s	O 1s	S 2p	N 1s	Normalized N 1s
Bare NH_2_-SAMs	36.9 (1.5)	53.6 (1.8)	4.6 (0.8)	1.6 (0.3)	3.3 (0.5)	-
Bare COOH-SAMs	43.7 (2.5)	45.9 (2.4)	9.0 (0.3)	1.8 (0.2)	-	-
CytoC on NH_2_-SAMs (100 μM)	25.2 (0.6)	57.7 (0.9)	8.8 (0.3)	1.1 (0.1)	7.2 (0.4)	6.6 (0.6)
CytoC on COOH-SAMs (100 μM)	25.3 (0.5)	55.5 (1.0)	10.5 (0.5)	1.0 (0.1)	7.6 (0.3)	-

Average experimental compositions were determined from two distinct samples (three spots per sample). All data were collected at a 0º TOA in the hybrid mode. Standard deviations are shown in parentheses.

As mentioned earlier, positively charged lysine residues are distributed around the surface of the protein [17]. Yet, on the end of the protein, opposite the heme group, there is a cluster of glutamic acid residues creating a region with no net surface charge. Previous work has demonstrated that on the COOH-SAMs, the heme group should be pointed towards the substrate [6]. Therefore, within the SIMS spectra collected from the CytoC-COOH-SAM samples, we should observe an enrichment of secondary ions from the leucine and glutamic acid residues. We hypothesize that on the NH_2_-SAMs, the heme group is pointed towards the vacuum interface. As a result, we should observe an enrichment of secondary ions from the cysteine residues.

**Figure 5:**
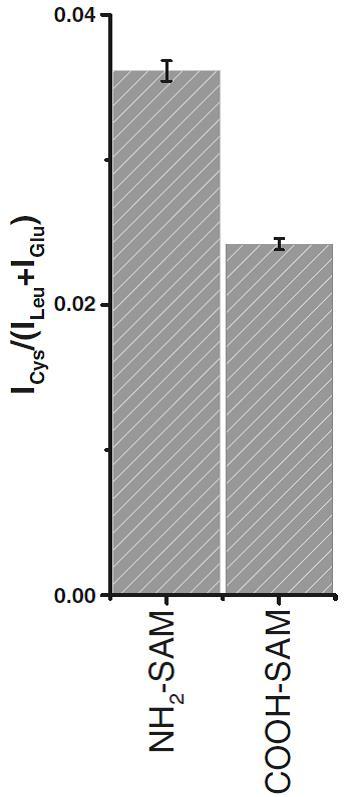
**ToF-SIMS peak intensity ratios calculated as the sum of intensities of secondary ions from cysteine (C**_**2**_**H**_**6**_**NS**^**+**^**) divided by the sum of intensities of secondary ions from glutamic acid (C**_**4**_**H**_**6**_**NO**^**+**^**, C**_**4**_**H**_**8**_**NO**_**2**_^**+**^**) and leucine (C**_**5**_**H**_**12**_**N**^**+**^**) residues.** Error bars represent the standard deviation across three analysis spots over two distinct samples.

To compare the orientations induced by the positive and negative substrates we compared the sum of the intensities of secondary ions stemming from cysteines to the sum of ions originating from leucines and glutamic acids. Figure 5 presents calculated intensity ratios for the two different samples, CytoC on NH_2_-SAMs versus CytoC on COOH-SAMs. Figure 5 illustrates significant changes in this ratio between CytoC on the two different SAM surfaces. As in previous investigations, the differences in this ratio can be interpreted as the protein adapting two opposite end-on protein orientations [18, 29, 30]. The 50% difference in the intensity ratios between the two different samples is less than the Protein G B1 electrostatic-immobilization scheme reported by Baio *et al.*[29] but similar to Baugh *et al.*[30] reported for the covalently-immobilized Protein G B1 model system.

The protein orientation inferred from the ToF-SIMS data agrees with previously published studies that showed when immobilized onto a COOH-SAMs electrode, the heme was pointed down towards the surface [6]. Previous electrochemical studies have demonstrated that the redox potential between the heme group and the electrode shifts when comparing CytoC immobilized onto COOH-SAMs versus NH_2_-SAMs of the same height [7]. Large differences in the reported ToF-SIMS ratios, across the two samples, also confirm that when CytoC interacts with a positively charged surface, this same dipole like distribution of charge induces the exact opposite orientation. As a result, the heme group is now pointing away from the surface, increasing the distance between itself and the substrate. The ToF-SIMS results illustrate that protein orientation can be directly related to electrochemical measurements.

## Conclusions

This investigation has taken advantage of the charge distribution around the protein to induce CytoC into two different orientations, thereby controlling the location of the heme group with respect to the substrate. SFG spectra collected from CytoC interacting with both negative and positively charged surfaces illustrate that electrostatic interactions drive the protein to form a well-ordered protein film. Observed differences in the intensity of secondary ions originating from amino acids asymmetrically distributed around the protein demonstrate that on the negatively charged substrate CytoC’s heme group is pointed down towards the substrate, consistent with previous studies of CytoC on COOH-SAMs. The secondary ion intensity differences also indicate that CytoC adopts an opposite orientation on the positively charged surface, which would point the heme group away from the substrate.
